# TβRII Regulates the Proliferation of Metanephric Mesenchyme Cells through Six2 In Vitro

**DOI:** 10.3390/ijms18040853

**Published:** 2017-04-18

**Authors:** Zhaomin Mao, Zhongshi Lyu, Liyuan Huang, Qin Zhou, Yaguang Weng

**Affiliations:** The M.O.E. Key Laboratory of Laboratory Medical Diagnostics, the College of Laboratory Medicine, Chongqing Medical University, Chongqing 400016, China; mao1204086118@163.com (Z.M.); 284003771@163.com (Z.L.); lyhuang0603@sina.com (L.H.); zhouqin@cqmu.edu.cn (Q.Z.)

**Keywords:** TβRII, Six2, Smad3, kidney development, proliferation

## Abstract

The transforming growth factor-β (TGFβ) family signaling pathways play an important role in regulatory cellular networks and exert specific effects on developmental programs during embryo development. However, the function of TGFβ signaling pathways on the early kidney development remains unclear. In this work, we aim to detect the underlying role of TGFβ type II receptor (TβRII) in vitro, which has a similar expression pattern as the crucial regulator *Six2* during early kidney development. Firstly, the 5-ethynyl-2′-deoxyuridine (EdU) assay showed knock down of *TβRII* significantly decreased the proliferation ratio of metanephric mesenchyme (MM) cells. Additionally, real-time Polymerase Chain Reaction (PCR) and Western blot together with immunofluorescence determined that the mRNA and protein levels of Six2 declined after *TβRII* knock down. Also, *Six2* was observed to be able to partially rescue the proliferation phenotype caused by the depletion of *TβRII*. Moreover, bioinformatics analysis and luciferase assay indicated Smad3 could transcriptionally target *Six2*. Further, the EdU assay showed that Smad3 could also rescue the inhibition of proliferation caused by the knock down of *TβRII*. Taken together, these findings delineate the important function of the TGFβ signaling pathway in the early development of kidney and *TβRII* was shown to be able to promote the expression of *Six2* through Smad3 mediating transcriptional regulation and in turn activate the proliferation of MM cells.

## 1. Introduction

The transforming growth factor-β (TGFβ) family signaling pathways are composed of various closely related proteins which share some structural homology but have separate receptors and take part in different functions; for example, activated TGFβ ligands bind to type 2 TGFβ receptor, which is a kinase, which recruits, phosphorylates, and activates the type 1 receptor that then phosphorylates receptor-regulated Smads to bind the co-Smads, acting as transcriptional factors [1]. This leads to the activation of different downstream target genes that function in many cellular processes, including proliferation, differentiation, apoptosis, cell growth, and other cellular functions both in embryo and adult organism [1,2].

During kidney development, there is a balance between consumption (differentiation) and self-renewal (proliferation) of Six2 positive mesenchymal nephron progenitor-cells (cap mesenchyme cells) in order to form the full complement of nephrons and nephron endowment. The former is instructed by the mutual inductive interactions from ureteric bud cells [3,4,5], which secrete Wnt9b, activating a canonical Wnt-β-catenin signaling pathway in cap mesenchyme cells [6,7,8,9]. The canonical Wnt pathway leads to the degradation of GSK-3β/CK1α/AXIN2/Adenomatous polyposis coli (APC) complex and the translocation of cytosolic β-catenin to nucleus, promoting extensive target genes including *Wnt4* and *Fgf8*. *Wnt4* and *Fgf8* expressed in the cap mesenchyme cells then trigger their differentiation (mesenchymal-to-epithelial transition (MET)) into an epithelial structure-renal vesicle [10,11], which subsequently gives rise to a single nephron. The development of nephron relies on Six2, with high expression in cap mesenchyme cells and low expression in renal vesicles, and is the crucial transcriptional regulator for promoting the proliferation of cap mesenchyme cells and inhibiting *Wnt4* and *Fgf8*-mediated differentiation of these cells. *Six2* knockout mice display ectopic differentiation, depletion of metanephric mesenchyme cells, and kidney hypoplasia and dysplasia [5,12].

Though Six2 serves an essential function in nephron progenitor cells maintenance during early kidney development, little is known about its underlying upstream regulation and related signal pathway. In this study, we showed that TGFβ type II receptor (TβRII) has a similar expression pattern with Six2 during kidney development, in line with prior RNA-sequencing research work [13] documenting that TβRII possesses a higher expression in the cap mesenchyme cells and a lower expression in renal vesicles. *TβRII* knock-down in metanephric mesenchyme (MM) cell line prevented the proliferation of metanephric mesenchyme cells and the phosphorylation of its downstream transcription factor *Smad3* as well as the expression of Six2 at mRNA and protein levels. Overexpression of Six2 was able to rescue the *TβRII* depletion-caused inhibition of proliferation phenotype. In addition, the TGFβ ligands activated the phosphorylation of Smad3 and enhanced the expression of Six2, while the inhibition of the TGF-β signal pathway by SB431542 decreased the expression of Six2 and suppressed the proliferation of mK3 cells. Further analysis proved that *Smad3*, activated by the TGF-β signaling pathway, transcriptionally regulated *Six2* and rescued the proliferation phenotype caused by the knockdown of *TβRII*. Our research revealed the functional effect of TβRII in metanephric mesenchyme cells and elaborated its specific molecular mechanism regarding Six2 up-regulation during kidney development.

## 2. Results

### 2.1. TβRII Promoted the Proliferation Rate of Metanephric Mesenchyme (MM) Cells

**Figure 1:**
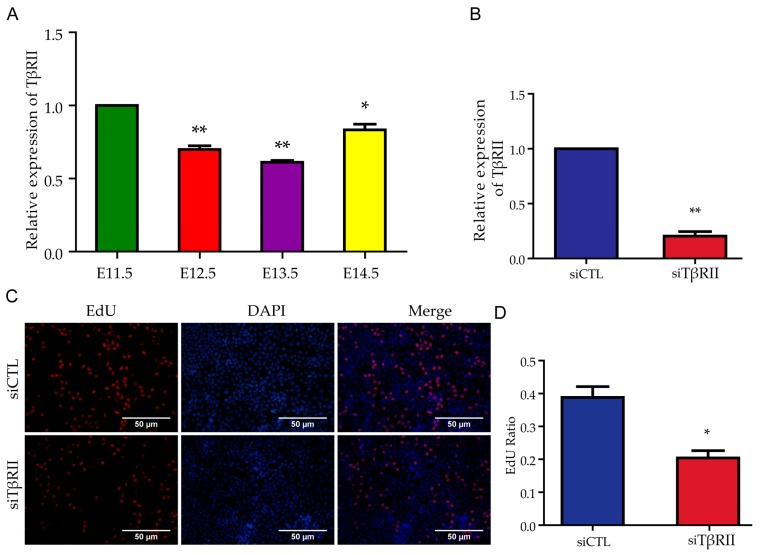
TGFβ type II receptor (*TβRII*) promotes the proliferation of mK3 cells. (**A**) The expression pattern of *TβRII* at mRNA level of embryo kidney from E11.5 to E14.5; (**B**) The mRNA level of *TβRII* in mK3 cells after the treatment of siTβRII; (**C**) The proliferation rate of mK3 cells after the transfection of siTβRII; (**D**) Histogram shows the quantitative analysis of the 5-ethynyl-2′-deoxyuridine (EdU) assay. All data are displayed as means ± Standard Deviation (SD) from three independent experiments, * *p* < 0.05, ** *p* < 0.01.

To explore whether TβRII was involved in early kidney development, we first detected the expression pattern of TβRII during early kidney development from E11.5 to E14.5. We found that *TβRII* exerted a relatively higher expression in E11.5 embryo kidney and a relatively lower expression after E11.5, which was in line with the RNA-sequence data [13] and indicated an important role of *TβRII* during early kidney development. We next transfected the siCTL (negative control with the sequence disorganized; 100 nM) and the siRNA of *TβRII* (siTβRII) (100 nM) in the mK3 cell lines [14]. Then we performed the real-time Polymerase Chain Reaction (PCR) to detect the expression of *TβRII* at mRNA level and found that the *TβRII* was significantly decreased (Figure 1B). Next, a 5-ethynyl-2′-deoxyuridine (EdU) assay was carried out to identify the proliferation rate of mK3 cells after the transfection, and the results showed that the proliferation rate was reduced by nearly half (Figure 1C,D). These data indicated that *TβRII* could promote the proliferation of MM cells.

### 2.2. TβRII Increased the Expression of Six2

**Figure 2:**
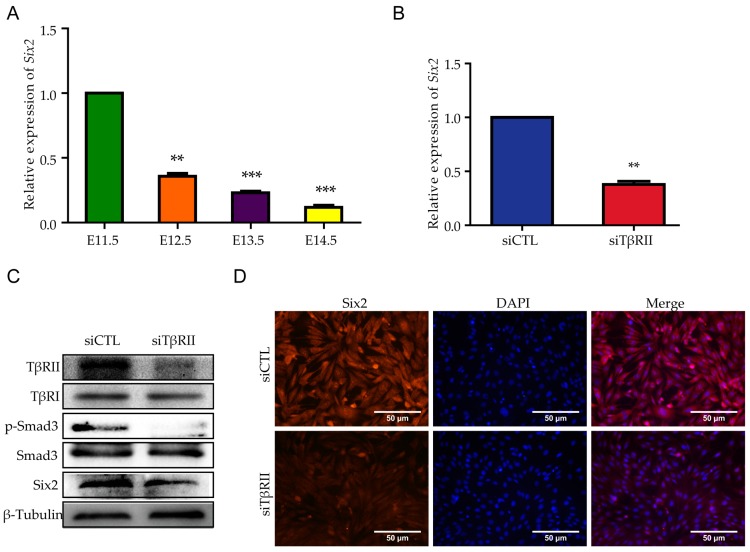
*TβRII* increases the expression of Six2 in mK3 cells. (**A**) The expression pattern of *Six2* at mRNA level of embryo kidney from E11.5 to E14.5; (**B**) The mRNA level of *Six2* in mK3 cells after the treatment of siTβRII; (**C**) The protein level of Six2, p-Smad3, Smad3, and TβRI in mK3 cells after the treatment of siTβRII; (**D**) The immunofluorescence of Six2 after the treatment of siTβRII. All data are displayed as means ± SD from three independent experiments, ** *p* < 0.01, *** *p* < 0.001.

Next, we explored the possible mechanism relating to the proliferation after the knockdown of *TβRII*. In order to investigate this problem, we first detected the expression pattern of *Six2*, which is one of the most classical regulators of MM proliferation, during early kidney development and found that the data was in line with the RNA-sequence data that showed a higher expression of *Six2* and *TβRII* in E11.5 embryo kidney and a lower expression of them in E12.5 embryo kidney (Figure 2A). After the discovery of the similar expression tendency, we wanted to explore the interrelationship between the data. Therefore, we transfected the siRNA (100 nM) into mK3 cells and first detected the expression of *Six2* by real-time PCR. The result demonstrated that the mRNA level of *Six2* decreased by more than 50% (Figure 2B). After the knock down of *TβRII*, the protein level of TβRII, Six2, and p-Smad3 were significantly decreased, while the expression of TβRI and Smad3 did not exhibit obvious change (Figure 2C). In line with the results above, the immunofluorescence also demonstrated a remarkable decline after the knock down of *TβRII* in mK3 cells (Figure 2D). These data suggested that TβRII could up-regulate the expression of Six2 both at mRNA level and protein level.

### 2.3. Overexpression of Six2 Can Rescue the Proliferation of MM Cells Induced by TβRII Deficiency

**Figure 3:**
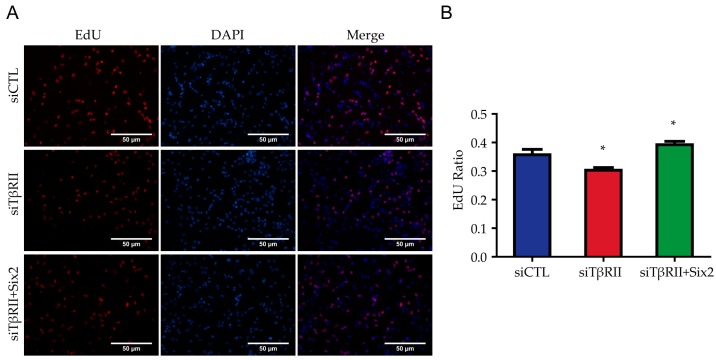
Six2 rescues the proliferation phenotype. (**A**) The proliferation rate of mK3 cells after the transfection of siTβRII and the co-transfection of *Six2*; (**B**) Histogram shows the quantitative analysis of the EdU assay above. All data are displayed as means ± SD from three independent experiments, * *p* < 0.05.

To further explore the important function of Six2 on the inhibition of proliferation caused by the knockdown of *TβRII*, we co-transfected siTβRII with *Six2* expression vector (pcDNA3.1(+)­Six2) into mK3 cells, and subsequently performed an EdU assay. As shown in Figure 3A,B, compared with the mK3 cells transfected with siTβRII and the control vector (pcDNA3.1(+)), co-transfection of siTβRII and pcDNA3.1(+)­Six2 can partially relieve the inhibited proliferation level. Therefore, Six2 overexpression could partially rescue the suppressed cell proliferation caused by deficiency of TβRII.

### 2.4. Smad3 Can Transcriptionally Regulate Six2 and Partially Rescue the Proliferation Phenotype

**Figure 4:**
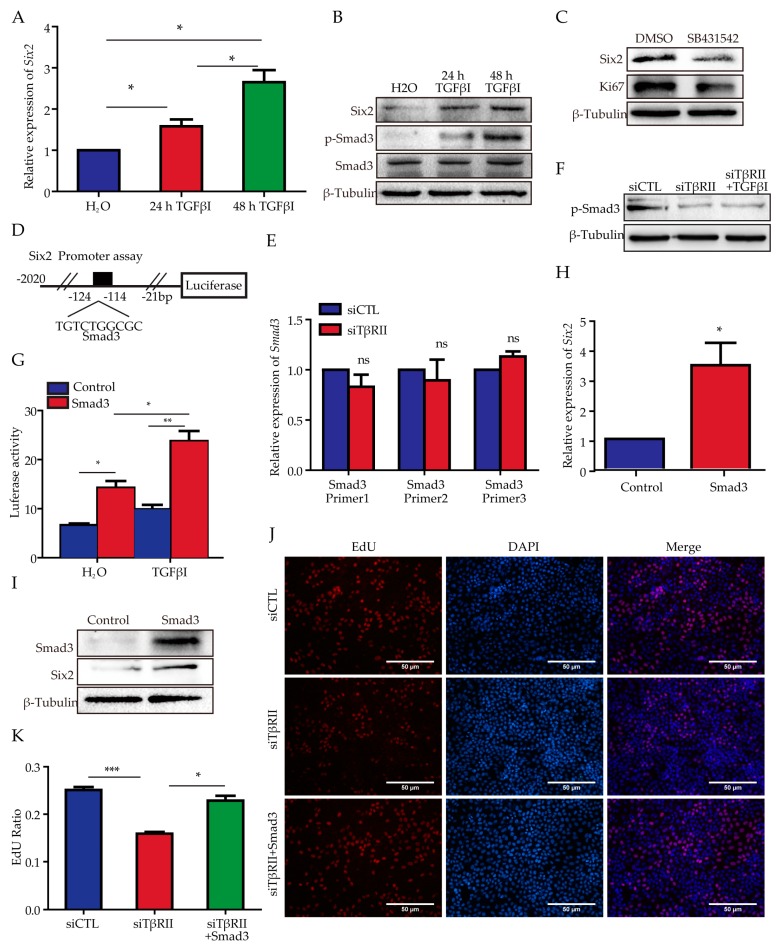
*Smad3* transcriptionally regulates *Six2* and rescues the proliferation phenotype. (**A**) The mRNA level of Six2 under the time course stimulation of TGFβI (2 ng/mL); (**B**) The protein levels of Six2 and p-Smad3 under the time course stimulation of TGFβI (2 ng/mL); (**C**) The protein level of Six2 and Ki67 after the treatment of phosphorylation Smad3 ( p-Smad3) inhibitor SB431542 (10 µM) for 48 h; (**D**) The promoter analysis of *Six2* via an online prediction tool (website: http://alggen.lsi.upc.es/cgi-bin/promo_v3/promo/promoinit.cgi?dirDB=TF_8.3); (**E**) The detection of the mRNA level of Smad3 via three pairs of primer in mK3 cells after the treatment of siTβRII; (**F**) The protein level of p-Smad3 with or without the stimulation of TGFβI (2 ng/mL) for 48 h after the knock down of *TβRII*; (**G**) pRL-SV40 and *Six2* promoter luciferase reporter together with the control vector or Smad3 under the stimulation of TgfβI (2 ng/mL) for 48 h were transfected into HEK293 cells. After 48 h, the cells were lysed and the fluorescences were detected and normalized to Renilla activity; (**H**) The mRNA level of *Six2* after the overexpression of Smad3 in mK3 cells; (**I**) The protein level of Six2 after the overexpression of Smad3 in mK3 cells; (**J**) The proliferation rate of mK3 cells after the transfection of siTβRII and the co-transfection of Smad3; (**K**) Histogram shows the quantitative analysis of the EdU assay above; (**K**) The proliferation rate of mK3 cells after the transfection of siTβRII and the co-transfection of Smad3. All data are displayed as means ± SD from three independent experiments. * *p* < 0.05, ** *p* < 0.01, *** *p* < 0.001.

To further confirm the function of the TGFβ signaling pathway towards *Six2*, we stimulated mK3 cells with TGFβI factor for 24 and 48 h, and the results demonstrated a gradual increase of *Six2* both at mRNA and protein levels, together with a significant increase of phosphorylation Smad3 (p-Smad3) (Figure 4A,B). The addition of p-Smad3 inhibitor SB431542 was shown to significantly decrease the expression of Six2 and suppress the expression of the proliferation maker Ki67 (Figure 4C). These data verified the conclusion that the activation of the TGFβ signaling pathway could promote the expression of *Six2* both at mRNA and protein levels.

In order to identify the specific transcription factor of *Six2*, we analyzed the promoter sequence of *Six2* (USSC) and searched the predicted transcriptional factor via PROMO version 2.0 (Universitat Politècnica de Catalunya, Barcelona, Spain) [15,16]. We focused on the predicted factor *Smad3* (from −114 to −124 bp) (TGTCTGGCGC) with the least dissimilarity (2.19%) (Figure 4D). Meanwhile, we detected the mRNA level of *Smad3* with three pairs of primer after the knockdown of *TβRII* and the result demonstrated no statistical significance (Figure 4E). The strength of TGFβ signaling in the absence of *TβRII* was assessed through the expression of p-Smad3, and the result demonstrated that knock down of *TβRII* could decrease the expression of p-Smad3 [17] and the addition of TGFβI factor did not result in an obvious increase of p-Smad3 (Figure 4F). Next, we conducted the luciferase assay to identify whether Smad3 could up-regulate the luciferase activity of the *Six2* promoter. The result showed that Smad3 could markedly increase the promoter activity, which indicated that Smad3 could promote the expression of Six2, and the stimulation of TGFβI could magnify the fold change compared with the cells transfected with Smad3 but without TGFβI stimulation (Figure 4G). Furthermore, the overexpression of Smad3 was able to increase both the mRNA and protein level of Six2 (Figure 4H,I). These data proved that Smad3 could transcriptionally regulate *Six2*. To detect the proliferation function of Smad3, we overexpressed Smad3 into the mK3 cells transfected with siTβRII. Compared with the mK3 cells transfected with siTβRII and pcDNA3.1(+), the co-transfection of siTβRII and pcDNA3.1(+)-Smad3 was able to partially rescue the proliferation phenotype (Figure 4J,K). All the data demonstrated that Smad3 can regulate *Six2* transcriptionally and partially relieve the inhibited proliferation.

## 3. Discussions

The development of kidney must balance the maintenance of renal progenitor cells (cap mesenchyme) and their differentiation into components of the mature nephron, the molecular mechanism of which is the opposing actions of β-catenin and Six2 in cap mesenchyme. Six2, the pluripotent renal cell regulator, plays a crucial role in maintaining the self-renew of cap mesenchymal cells, and deletion of Six2 will disrupt the balance of nephron progenitors between self-renew and differentiation [5,6,18]. These research studies implied that the research of the upstream regulatory factor towards Six2 is of great importance. According to the limited associated reports, the miR181 family is able directly target the 3′UTR of *Six2* and suppress its expression [18,19], Recombination signal binding protein for immunoglobulin kappa J region (Rbpj) is sufficient for the down-regulation of Six2 [20], Notch2 can shut down the Six2 mediated program for progenitor maintenance [21], and Pax2/Eya1/Hox11 complex binds to the proximal promoter of *Six2* and thus regulates its expression [22].

Recently, TβRII was shown to have a similar expression pattern with *Six2*, the crucial transcriptional factor maintaining the self-renewal of the cap mesenchyme, between cap mesenchyme (CM) and the renal vesicles (RVs). To define the effect and underlying mechanism of TβRII during kidney development specifically, we inhibited the expression of *TβRII* via siRNA in mK3 cell line and found that *TβRII* promoted the proliferation of mK3 cells and *Six2* expression, which can be rescued by *Six2* overexpression. Further bioinformatics analysis predicted the putative binding sites of *Smad3* in the promoter region of *Six2*. A subsequent luciferase assay verified that Smad3 could transcriptionally target *Six2*. Furthermore, the role of Smad3 in the TGFβ signaling pathway in mK3 cells was also confirmed by the rescue assay of mK3 cells’ proliferation experiment.

## 4. Materials and Methods

### 4.1. Cell Culture and Transfection

Human Embryonic Kidney 293 (HEK293) and mK3 cells were cultured in Dulbecco’s Modified Eagle’s Medium (DMEM)(Gibco, Carls-bad, CA, USA) supplemented with 10% fetal bovine serum (Gemini, Shanghai, China), penicillin, and streptomycin (Gibco) at 37 °C, 5% CO_2_, and 100% humidity. All the plasmids and siRNA (GenePharma, Shanghai, China) (the sequence is listed in Table S1) were transfected via Lipofectamine^TM^2000 (Invitrogen, Carlsbad, CA, USA) according to the manufacturer’s instructions.

### 4.2. Plasmid Construction

The coding sequence (CDS) of *Six2* was amplified via polymerase chain reaction (PCR) from the cDNA of mK3 cells and cloned at the site of BamHI and EcoRI to generate pcDNA3.1(+)-Six2. The CDS of Smad3 was amplified via PCR from HEK293 cells and cloned into pcDNA3.1(+). The promoter of *Six2* was amplified from C57BL/6 genomic DNA and inserted into the upstream of luciferase coding sequence of pcDNA3.1-luciferase reporter vector. The primer information is provided in Table S2.

### 4.3. Luciferase Reporter Assay

Luciferase reporter assays were performed as previously described [18].

### 4.4. Collection of Embryo Kidney

The morning of the discovery of the vaginal plug was considered as E0.5. We waited until E11.5, E12.5, E13.5, and E14.5, collected the kidneys of these embryos, and stored them at −80 °C.

### 4.5. Real-time Polymerase Chain Reaction (PCR)

Total embryo kidney RNA and mK3 cellular RNA were extracted with TRIzol (Invitrogen) and the RNA was reverse-transcribed using the First-Strand cDNA Synthesis kit (Thermo Scientific, Walham, MA, USA) following the manufacturer’s instructions. The quantitative PCR reactions were conducted using UltraSYBR Mixture (CWBIO, Guangzhou, China). The expression levels of *Six2*, *Smad3*, and *TβRII* were normalized to that of 18 s. The primers used are listed in Table S2.

### 4.6. Western Blot

The mK3 cells were collected 48 h after the transfection. The proteins were extracted following the process as previously described [18]. The membranes were incubated with primary antibodies (mouse anti-β-Tubulin (1:2000 dilution, Cell Signaling Technology, Beverly, MA, USA); rabbit anti-Six2 (1:1000 dilution, Proteintech, Wuhan, China); rabbit anti-TβRII (1:300 dilution, Boster, Wuhan, China); rabbit anti-TβRI (1:500 dilution, Proteintech); rabbit anti-Smad3 (Cell Signaling Technology); rabbit anti-p-Smad3 (Abcam, Cambridge, MA, USA) at 4 °C overnight. The secondary antibodies were goat anti-rabbit Immunoglobulin G (IgG) and goat anti-mouse IgG (1:5000 dilution, Proteintech). The final signals were developed by Chemiluminescent Horseradish Peroxidase (HRP) Substrate Reagent (Millipore, Billerica, MA, USA) and were detected with ChemiDoc™ XRS+ (Bio-Rad, Hercules, CA, USA).

### 4.7. Drug Stimulation

The mK3 cells and HEK 293 cells were treated with TGFβI factor (Novoprotein, Shanghai, China) (dissolved in sterile double distilled H_2_O (ddH_2_O)) with the final concentration of 2 ng/mL for 24 h or 48 h. The mK3 cells were treated with SB431542 (MedChem Express, Monmouth Junction, NJ, USA) (dissolved in sterile Dimethylsulfoxide (DMSO)) with the final concentration of 10 µM for 48 h.

### 4.8. Immunofluorescence

Forty-eight hours after the transfection, the mK3 cells were fixed with 4% paraformaldehyde at room temperature for 20 min, then washed with Phosphate Buffer Solution (PBS) for 5 min three times and blocked with 10% goat serum with 0.1% Triton X-100 in PBS at room temperature for 1 h. Afterwards, the cells were incubated with the primary antibody (rabbit anti-Six2, 1:100 dilution, Proteintech) at 4 °C overnight. The cells were washed three times with PBS for 5 min and incubated with the secondary antibody (goat anti-rabbit FITC, 1:500 dilution, Invitrogen) at room temperature for 1 h away from light. Next, they were dyed with 4′,6-diamidino-2-phenylindole (DAPI) for 10 min at room temperature. The map was detected by fluorescence microscopy.

### 4.9. EdU Cell Proliferation Assay

5-Ethynyl-2′-deoxyuridine (EdU) assay was conducted to detect the cell proliferation. Forty-eight hours after transfection, the proliferation experiment of the mK3 cells was performed with Cell-Light EdU Apollo 567 In Vitro Kit (RiboBio Co., Ltd., Guangzhou, China) according to the manufacturer’s instructions. The final proliferation rate of mK3 cells was determined by the ratio of EdU-staining-positive cells (red) to the total cell number labelled by DAPI (blue).

### 4.10. Statistical Analysis

The software of GraphPad Prism 5 (GraphPad Software, Inc., La Jolla, CA, USA) was used to analyze all the data. All data were collected from at least three independent experiments. The results were shown as means ± SD with *p* < 0.05 considered statistically significant.

## 5. Conclusions

**Figure 5:**
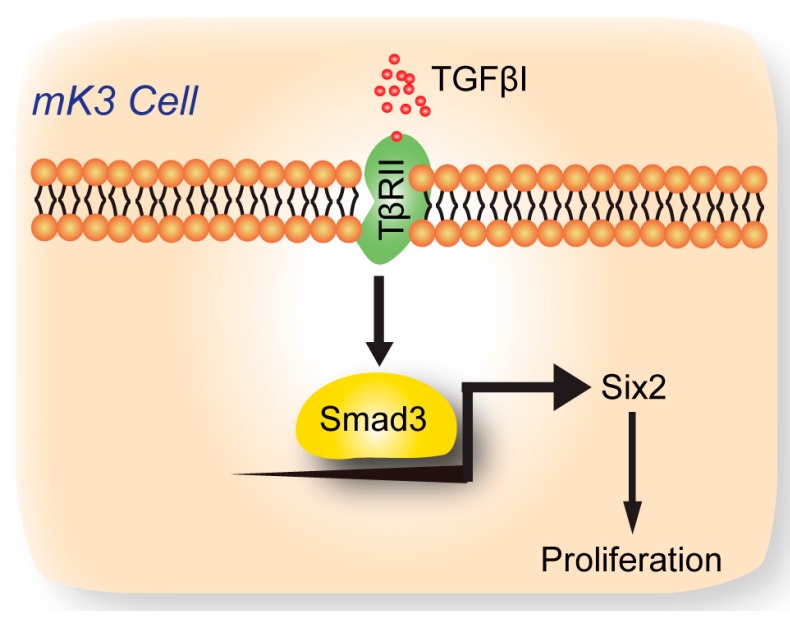
A schematic diagram of the molecular mechanism that TβRII promotes the proliferation of metanephric mesenchyme (MM) cells.

Taken together, our research described four important findings (Figure 5): (1) That knockdown of TβRII could inhibit the proliferation of mK3 cells; (2) that the expression of Six2 was inhibited after the knockdown of TβRII; (3) that TβRII regulated the proliferation of MM cells through Six2; (4) that the activation of the TGFβ signaling pathway increased the expression of *Six2*; (5) that TβRII regulated Six2 through the transcriptional regulation of Smad3. This mechanism was observed in the mK3 cells, and the specific role of TβRII in early kidney development in vitro was verified by Six2. Given the critical role of TβRII in early kidney development, we will continue to explore whether TβRII can affect the embryonic kidney development in vivo. The insights from the recent and ongoing studies could potentially lead to the confirmation of the specific role of TGFβ and TβRII in early kidney development.

## Supplementary Materials

Supplementary materials can be found at www.mdpi.com/1422-0067/18/4/853/s1.
